# Early Laparoscopic Cholecystectomy with Continuous Pressurized Irrigation and Dissection in Acute Cholecystitis

**DOI:** 10.1155/2015/734927

**Published:** 2015-02-24

**Authors:** I. Ozsan, O. Yoldas, T. Karabuga, U. M. Yıldırım, H. Y. Cetin, O. Alpdoğan, U. Aydin

**Affiliations:** ^1^Department of General Surgery, Faculty of Medicine, İzmir University, 1825 Sokak Yeni Girne Mh., Karșiyaka, 35520 İzmir, Turkey; ^2^Department of Radiology, Faculty of Medicine, İzmir University, 1825 Sokak Yeni Girne Mh., Karșiyaka, 35520 İzmir, Turkey; ^3^Department of Anesthesiology and Reanimation, Faculty of Medicine, İzmir University, 1825 Sokak Yeni Girne Mh., Karșiyaka, 35520 İzmir, Turkey

## Abstract

*Background*. The aim of this study was to evaluate the preliminary results of a new dissection technique in acute cholecystitis.* Material and Method*. One hundred and forty-nine consecutive patients with acute cholecystitis were operated on with continuous pressurized irrigation and dissection technique. The diagnosis of acute cholecystitis was based on clinical, laboratory, and radiological evidences. Age, gender, time from symptom onset to hospital admission, operative risk according to the American Society of Anesthesiologists (ASA) score, white blood cell count, C-reactive protein test levels, positive findings of radiologic evaluation of the patients, operation time, perioperative complications, mortality, and conversion to open surgery were prospectively recorded.* Results*. Of the 149 patients, 87 (58,4%) were female and 62 (41,6%) were male. The mean age was 46.3 ± 6.7 years. The median time from symptom onset to hospital admission 3.2 days (range, 1–6). There were no major complications such as bile leak, common bile duct injury or bleeding. Subhepatic liquid collection occurred in 3 of the patients which was managed by percutaneous drainage. Conversion to open surgery was required in four (2,69%) patients. There was no mortality in the study group.* Conclusion*. Laparoscopic cholecystectomy with continuous pressurized irrigation and dissection technique in acute cholecystitis seems to be an effective and reliable procedure with low complication and conversion rates.

## 1. Introduction

Acute cholecystitis is an acute inflammatory disease of the gallbladder caused by bacterial infection and biliary tract obstruction [1, 2]. In the first decade of laparoscopic era acute cholecystitis (AC) was considered a contraindication for a minimally invasive approach, but, nowadays, laparoscopic cholecystectomy (LC) is indicated as the treatment of the choice for patients with AC and recent meta-analyses suggest that early laparoscopic cholecystectomy is advantageous [3–5]. The tissues are friable, the inflammatory changes make the dissection difficult and surgical planes are ill-defined in acute cholecystitis, and laparoscopic cholecystectomy becomes more difficult and potentially more dangerous. In order to facilitate the dissection of edematous and inflamed tissues and to decrease the possibility of complications, a new technique of laparoscopic cholecystectomy with continuous pressurized irrigation and dissection for acute cholecystitis was defined.

## 2. Materials and Method 

One hundred and sixty-two patients were admitted to İzmir University, Faculty of Medicine Hospital, with the diagnosis of acute cholecystitis between December 2011 and December 2013. 13 patients underwent open cholecystectomy because of preoperative evidence or suspect of gallbladder perforation, diffuse peritonitis, and being excluded from the study. One hundred and forty-nine consecutive patients, 12 of them having a history of previous upper abdominal surgery, with acute cholecystitis were operated on with continuous pressurized irrigation and dissection technique. Acute cholecystitis was diagnosed in patients if at least three of the following four criteria were detected: elevated white blood cell count (WBC > 10,000/mm^3^), right hypochondriac tenderness, fever higher than 38°C, and radiologic evidence of AC. Gallbladder wall thickening (>5 mm), probe tenderness, and pericholecystic fluid were the radiologic evidence of acute cholecystitis. Laparoscopic cholecystectomy was performed in the first 24 hours of admission to the hospital in all patients. All the patients received parenteral third-generation cephalosporin preoperatively. All patients were operated on by or under the supervision of the same surgeon (UA).

### 2.1. Operative Technique

**Figure 1:**
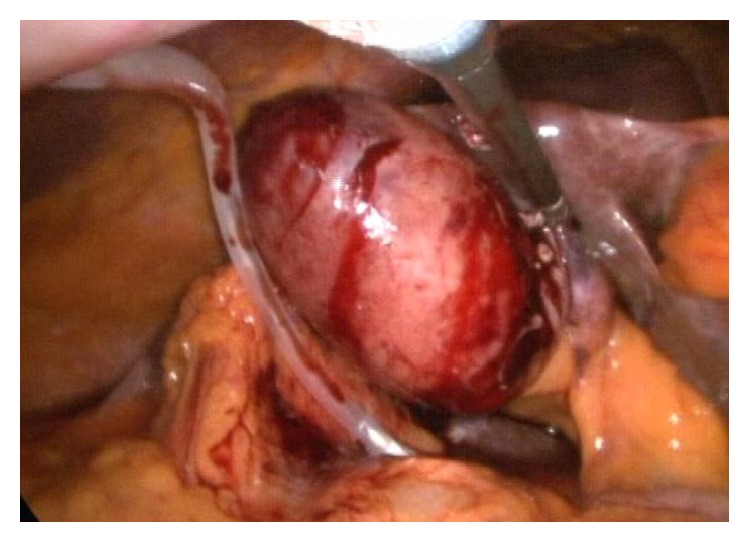
The image shows the replacement of drain from the fourth trocar just before starting the Calot dissection to provide the suction of the water from dissection area.

**Figure 2:**
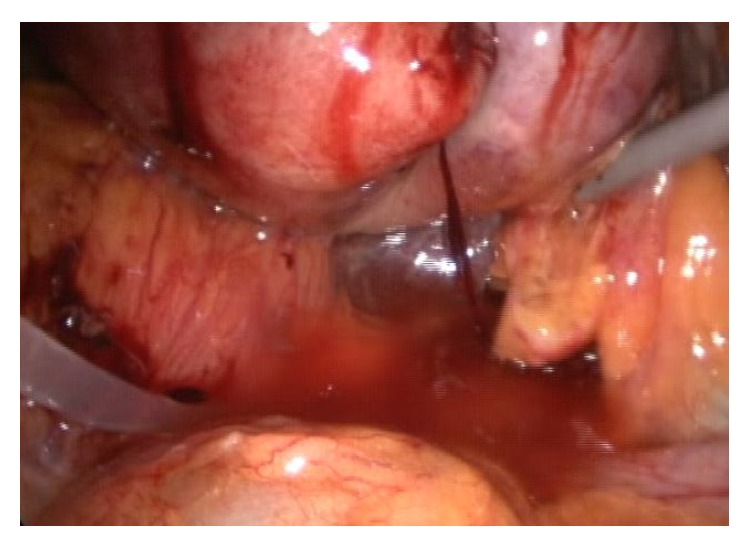
The image shows the pressurized irrigation and blunt dissection of the Calot triangle.

**Figure 3:**
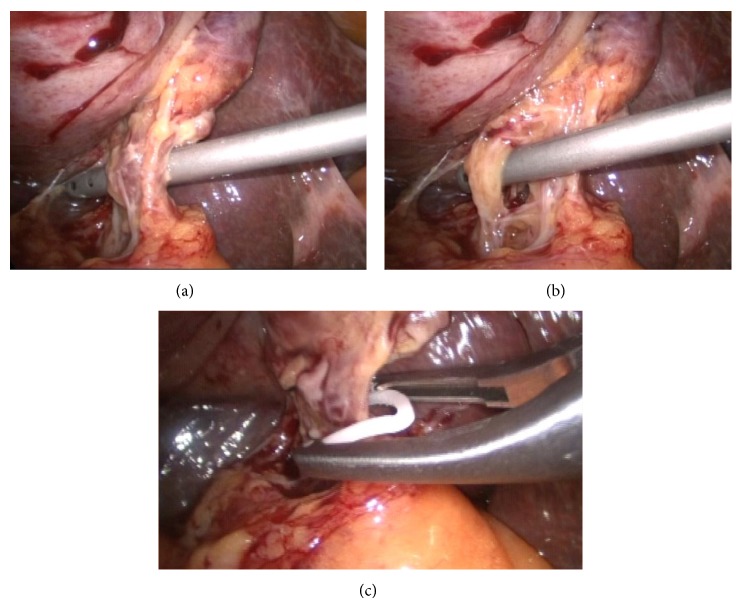
(a) The image shows the isolation of the cystic duct after dissection of the Calot triangle. (b) The image shows the isolation of cystic artery after dissection of the Calot triangle, and cystic artery and the cystic duct are isolated separately before clipping. (c) The image shows the clipping the cystic duct with hem-o-lok clip.

**Figure 4:**
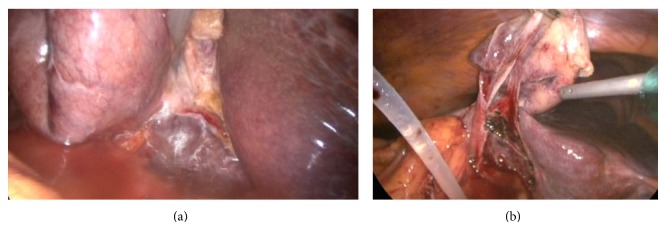
(a) The image shows the pressurized irrigation and dissection of the gallbladder from the liver after transection of the cystic artery and the cystic duct. (b) The image was obtained just before the removal of the gallbladder at the end of the pressurized irrigation and dissection.

Laparoscopic cholecystectomy was performed through a three-trocar operative technique (using a 10-mm optical trocar at the infraumbilical region, a 10-mm operating trocar at the infraxiphoid region, and a 5-mm operative trocar at the right subcostal region on midclavicular line) with angled (30°) laparoscope. Entrance to the abdomen was achieved with video-assisted trocar. Instead of the fourth trocar a drain was replaced from right subcostal-anterior axillary line through the subhepatic space to drain off the irrigation solution during the operation (Figure 1). The drain was clamped and opened intermittently to avoid the accumulation of the irrigation solution during gallbladder dissection. After gallbladder traction from Hartmann's pouch, the blunt dissection of Calot's triangle was achieved by a metal irrigator with continuous pressurized irrigation (CPI) (Figure 2). Pressurizing of the irrigation solution was carried out by using pressure infusion cuff (P.J. Dahlhausen & Co. Gmbh) at a standard pressure of 200 mmHg. Either the dissection of edematous plan between gallbladder and liver or the bloodless dissection area was provided by continuous pressurized irrigation and blunt dissection with metal irrigator (Figures 4(a)-4(b)). Neither dissectors nor hooks were used until the isolation of cystic artery and duct (Figures 3(a)-3(b)). Cystic artery and duct were clipped by hem-o-lok polymer ligation clips (Weck/Teleflex) (Figure 3(c)). The gallbladder was extracted from the epigastric port incision in an endoscopic bag in all patients. The closed suction drain was left after operation on all patients.

### 2.2. Postoperative Care and Data Collection

All patients were mobilized 4 hours after the operation and were put on liquid diet on postoperative sixth hour. Third-generation 1 g cephalosporin was administered twice a day during the hospitalization. The drains were removed when the drainage decreased to ≤50 mL/day. All patients were discharged after removal of the drain with antibiotics and anti-inflammatory drugs.Patients were examined routinely on the surgical ward on postoperative days 3 and 10 for wound inspection and removal of sutures. Age, gender, time from symptom onset to hospital admission, operative risk according to the American Society of Anesthesiologists (ASA) score, white blood cell counts, C-reactive protein test levels, positive findings of radiologic evaluation of the patients, operation time, perioperative complications, and conversion to open surgery were prospectively recorded.

## 3. Results

Of the 149 patients, 87 (58.4%) were female and 62 (41.6%) were male. The mean age of the study group was 46.3 ± 6.7 (range, 28–73). The median time from symptom onset to hospital admission was 3.2 days (range, 1–6). The median values of WBC and C-reactive protein were 13,200/mm^3^ (9100–19700) and 8.9 mg/dL (3.1–21.3), respectively, at initial hospital admission. The distribution of American Society of Anesthesiologists (ASA) score among patients is as follows: 69 (46.3%) were in ASA III, 46 (30.9%) were in ASA 2, 23 (15.4%) were in ASA 1, and 11 (7.4%) were in ASA 4. Preoperative radiologic examination revealed gallbladder wall thickness in 83.4% of the patients, hydropic gallbladder in 67.3%, and probe tenderness (ultrasonographic Murphy sign) in 48.4%. Five patients underwent preoperative endoscopic retrograde cholangiopancreatography (ERCP) because of coexisting common bile duct stones. All five patients had had cholecystectomy on the same day of ERCP. The mean operation time was 67.8 ± 19.3 min (range, 32–116). There were no major complications such as bile leak, common bile duct injury, or bleeding. Subhepatic liquid collection occurred in 3 of the patients (2.01%) which was managed by percutaneous drainage. Conversion to open surgery was required in four (2.69%) patients. Three of these four patients were having a history of previous upper abdominal surgery and the conversion reason was the severe peritoneal adhesions. The reason of conversion for the remaining one patient was dense adhesions around the gallbladder. Conversion to open cholecystectomy was not required due to the inability to demonstrate the anatomic structures of Calot's triangle. There was no mortality in the study group. The mean duration of hospital stay was 3.93 days in the study group.

## 4. Discussion

Laparoscopic cholecystectomy performed for acute cholecystitis is technically difficult due to inflammatory adhesions and distortion of biliary anatomy. Although early laparoscopic cholecystectomy is shown to be safe, feasible, and associated with a shorter hospital stay in some randomized trials [6, 7], it is associated with high rates of conversion to open cholecystectomy and an increased incidence of complications [8–10]. The most common cause of conversion was difficulty of exhibiting the anatomical structures in Calot's triangle [11–15]. However, with growing experience and greater technical skills, surgeons realized that these obstacles could be managed. Consequently, an increasing number of reports became available, demonstrating the feasibility of the laparoscopic approach for acute cholecystitis with an acceptable morbidity [16, 17].

Failure of the conservative treatment, recurrence of acute cholecystitis before interval surgery, and the need for two hospital admissions are the disadvantages of delayed surgery. In addition the patients managed conservatively and receiving delayed laparoscopic cholecystectomy has a high risk (up to 17.5%) of an emergency interval procedure with a 45% conversion rate to open cholecystectomy [18]. Laparoscopic dissection is shown to be difficult and unsafe due to the presence of dense fibrous adhesions in delayed laparoscopic cholecystectomy [7]. The main reason for a conservative approach was the concern of having a high risk of common bile duct injury due to edematous and inflamed tissues obscuring the anatomy in Calot's triangle. Bile duct injury can sometimes even be fatal because of sepsis and the corrective surgery for bile duct injury also carries morbidity and mortality.

High conversion rates have been reported with the presence of pericholecystic fluid or edema and thickening of the gallbladder wall [19]. Prakash et al. also reported that 13 of 24 (56.5%) patients with preoperative evidence of pericholecystic fluid collection on ultrasound underwent conversion to open surgery [20]. However, in our study, neither gallbladder wall thickening nor pericholecystic fluid was the reason for conversion to open surgery. Conversion and complication rates are the indicators of the safety, cost-effectiveness, and acceptance of a procedure. Taking into consideration the results of the present study, laparoscopic cholecystectomy with continuous pressurized irrigation and dissection technique seems to be an effective and reliable procedure with low complication and conversion rates.
